# Oblique coronal view of the ACL double-bundle: Comparison of the Chinese Visible Human dataset and low-field MRI

**DOI:** 10.3892/etm.2013.1169

**Published:** 2013-06-20

**Authors:** WEI CHEN, BING XIE, GUO-HONG ZU, GERIGK LARS, LI-WEN TAN, JIAN WANG, SHAO-XIANG ZHANG, YA-MING WEN, YONG-KE ZHANG, LING CHEN

**Affiliations:** 1 Department of Radiology, Southwest Hospital, The Third Military Medical University, Chongqing 400038, P.R. China; 2 Department of Radiology, Jinan Central Hospital Affiliated to Shandong University, Jinan, Shandong 250013, P.R. China;; 3 Division of Radiology, German Cancer Research Center, Heidelberg 69120, Germany;; 4 Department of Anatomy, College of Medicine, The Third Military Medical University, Chongqing 400038, P.R. China

**Keywords:** anterior cruciate ligament, anatomy, magnetic resonance imaging, oblique coronal plane, Chinese Visible Human

## Abstract

The aim of this study was to distinguish the individual bundles of the anterior cruciate ligament (ACL) using the Chinese Visible Human (CVH) dataset and images obtained by low-field routine magnetic resonance imaging (MRI) in the oblique and coronal planes. Sectional anatomical data of the knee were selected from the CVH dataset and reconstructed in 3D. MRI of normal knees was performed with a low-field-strength magnet in the coronal plane. The shape of the ACL was clearly displayed. Using the oblique coronal plane, the anteromedial (AM) and posterolateral (PL) bundles of the ACL were distinguished in the reconstructed anatomical data and the MR images. The double-bundle structure of the ACL was evaluated in the CVH 3D reconstructions and MR images. Using the oblique coronal plane, it was possible to review the ACL structure in the knee. The study demonstrated the feasibility of distinguishing the two bundles in the ACL with CVH 3D reconstruction and low-field strength MRI. The accuracy in the grading of ACL injury in presurgical planning may be improved.

## Introduction

The anterior cruciate ligament (ACL), which resists anterior tibial translational and rotational loads, is a key structure in the knee joint and one of the most frequently injured in clinical practice (1). Magnetic resonance imaging (MRI) is the preferred method for the diagnosis of ACL and associated injuries, and has been demonstrated to be highly accurate in the assessment of the native ACL and complete ACL tears. However, the sensitivity and specificity of MRI for the detection of partial ACL tears is low. Grading the injury using MR images is of benefit when planning ACL reconstruction surgery, since partial tears do not always require surgical reconstruction. If only one bundle is predominantly torn, isolated single bundle reconstruction, rather than full ACL graft reconstruction, may be undertaken. The grading of ACL tears describes the rupture pattern more precisely. This may enable surgical planning to be improved and allow reconstruction of the two bundles based on their individual status. The early evaluation of partial ACL tears may improve clinical and surgical management. Anatomical studies have shown that the ACL consists of two distinct functional bundles, termed the anteromedial (AM) and posterolateral (PL) bundles, based on their tibial insertions (2). The double-bundle model of the ACL is widely accepted. Studies of biomechanics and kinematics have demonstrated that each bundle is important in the stability of the knee. The AM and PL bundles have distinct biomechanical functions, with the AM bundle stabilizing the knee in flexion and the PL bundle stabilizing the knee in extension (3,4). The double-bundle reconstruction technique (DBT) aims to restore the function of the AM and PL bundles (5). A prerequisite for diagnosing ACL bundle injury using MR imaging is a clear delineation of the two bundles and an awareness of their normal appearance. Routine MRI examination planes are able to delineate the normal ACL bundle structure in only 42% of knees (6). The value of standard planes to distinguish injuries of the AM and PL bundles is limited and the identification of an ideal combination of slice orientation, thickness and pulse sequences remains a key area of investigation (7,8). In the current study, we describe an oblique coronal plane for imaging the normal ACL bundles using low-field strength MRI at 0.2 T and evaluate the efficacy with which the normal ACL bundles may be evaluated on this plane compared with 3D reconstructions of the Chinese Visible Human (CVH) dataset.

## Subjects and methods

### Subjects

The study population was selected during the period from September 2004 to January 2007. This study was conducted in accordance with the Declaration of Helsinki and with approval from the Ethics Committee of the Southwest Hospital of the Third Military Medical University (Chongqing, China). Written informed consent was obtained from all participants. MR images were obtained from 120 knees according to the imaging protocol. The inclusion criteria for the study population consisted of completed growth (closed epiphyseal plates), an age of ≤50 years and no previous injury or knee surgery. All knee MR images were reviewed by three radiologists, and only knees with an intact cruciate ligament were included. Knees that displayed evidence of notch hypoplasia or stenosis, or partial or complete ACL or PCL deficiencies were excluded. The study population consisted of 120 individuals (60 males and 60 females; age range, 22–48 years; mean age, 34 years), who were divided into four groups: left and right knee in female or male patients.

MRI was performed with a low-field 0.2 T Artoscan MRI scanner (Esaote S.p.A., Genova, Italy), with the knee held in full extension. Images of the knee were typically obtained in the axial, oblique sagittal and oblique coronal planes, with the coronal plane oriented parallel to a line drawn through the epicondyles. The oblique sagittal images were planned on axial images and oriented at 90° to the coronal images. The MRI protocol consisted of an oblique sagittal T1-weighted spin echo [repetition time/echo time (TR/TE)=840/26 msec]; a T2-weighted turbo spin echo [TR/TE=3,000/80 msec; echo train length (ETL), 4; slice thickness, 4 mm; interval, 0.2 mm; matrix, 256×256; field of view (FOV), 12 cm] and a double echo with T2 and proton density-weighted images and coronal T2-weighted gradient echo (TR/TE=480–560/16–20 msec, flip angle, 400). The oblique coronal slices were planned to be parallel to the course of the femoral intercondylar roof on a sagittal scout image and acquired in T2-weighting (TR/TE=3,000–4,000/96 msec; ETL, 2; slice thickness, 2–3 mm; interval, 0.2–0.3 mm; matrix, 256×125) and T1-weighting (TR/TE=540–600/28 msec).

For the coronal, sagittal and oblique images, each observer was asked to evaluate whether they were able to distinguish the AM and PL bundles at the femoral, mid-substance and tibial levels. Each MRI was independently read in a blinded fashion by a total of three observers: one musculoskeletal fellowship-trained radiologist, one senior orthopedic resident and one junior orthopedic resident. The time between observations for each reviewer was at least 4 weeks.

### Cryosection of specimens

Following the acquisition of approval from the Institutional Ethical Committee, middle-sized male cadavers without organic lesions, as verified by visual inspection, were examined. Subsequent to vascular perfusion, the specimens were embedded in gelatin and then placed in a −30°C saline pool for cryopreservation for 1 week, prior to being stored in the laboratory at −25°C. The specimens were serially sectioned from head to foot (sectioning accuracy: 0.0001 mm) using a TK-6530 Digital Sectioner (Hanzhong Machine Tool Factory, Hangzhou, China). The slice thicknesses were 0.5 and 0.25 mm. Photographic images of all slices were serially captured using a digital camera (Canon, Tokyo, Japan) with a resolution of 3,072×2,048 and each sectioned image file of 36 MB was stored on a personal computer (PC).

### Computerized 3D reconstruction

Prior to embedding, four plastic rods were placed in the mold parallel to the long axis of the cadaver for use as a reference for the alignment of the computerized slices. The alignment was performed on a PC. Using the commercially available Amira^®^ 3D reconstruction software (Visualization Sciences Group, Boston, MA, USA), the single slices were merged into a 3D dataset. The 2D images of the ACL were reconstructed in sagittal, coronal and oblique coronal sections. On the cryospecimen, the orientation of the coronal oblique plane was defined along the true anatomical course of the ACL. This was achieved by adjusting the angle of the cryosectional plane parallel to the femoral intercondylar roof. The length and the angle of the ACL were measured on the oblique sagittal planes. The statistical analysis was performed using standard software (SPSS for Windows, version 10.0; SPSS, Inc., Chicago, IL, USA). P<0.05 was considered to indicate a statistically significant result.

## Results

### Measurement of the ACL

The structure of the two ACL bundles was reviewed on the MR images. The two bundles of the ACL were not clearly distinguishable on the oblique sagittal or coronal planes. For the left and right knee groups, the length, width and coronal angle of the ACL are summarized in Table I. The ideal angulations of the oblique coronal slices were obtained from an average coronal angle of the ACL. There were no significant differences in the length and thickness of the ACL between the left and right knee groups; however, significant differences were observed in the length, thickness and coronal angles between males and females.

### AM and PL bundles

**Figure 1.**
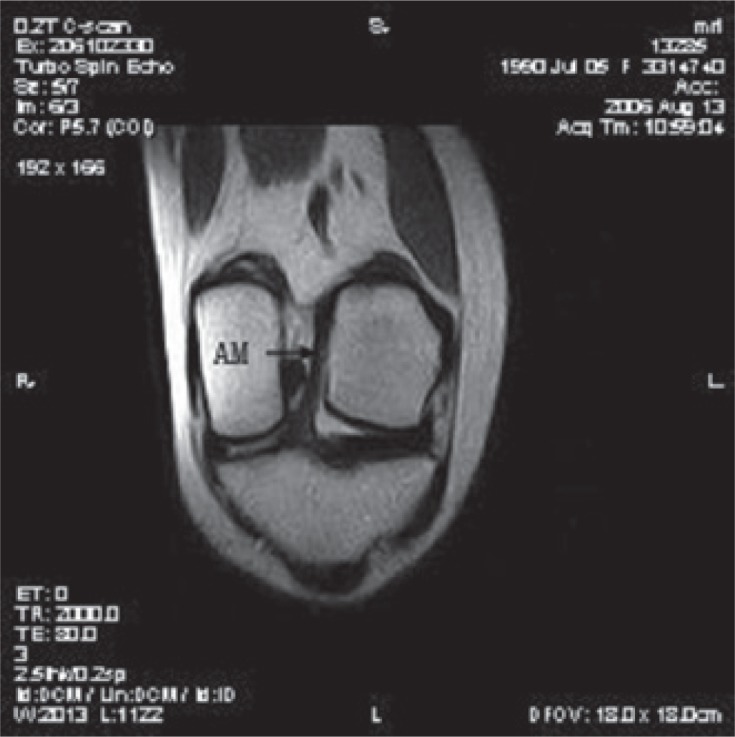
3D reconstruction using the oblique coronal plane showing the anteromedial (AM) bundle (arrow).

**Figure 2.**
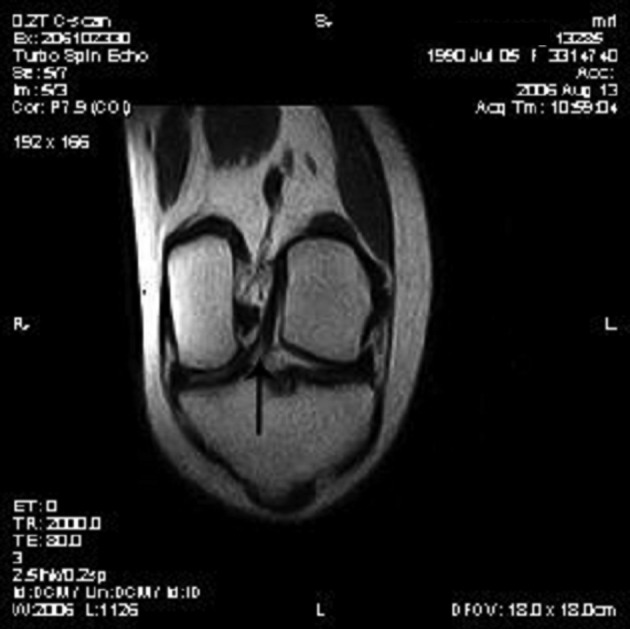
3D reconstruction using the oblique coronal plane showing the anteromedial (AM) and posterolateral (PL) bundles with a fibrofatty septum (arrow).

**Figure 3.**
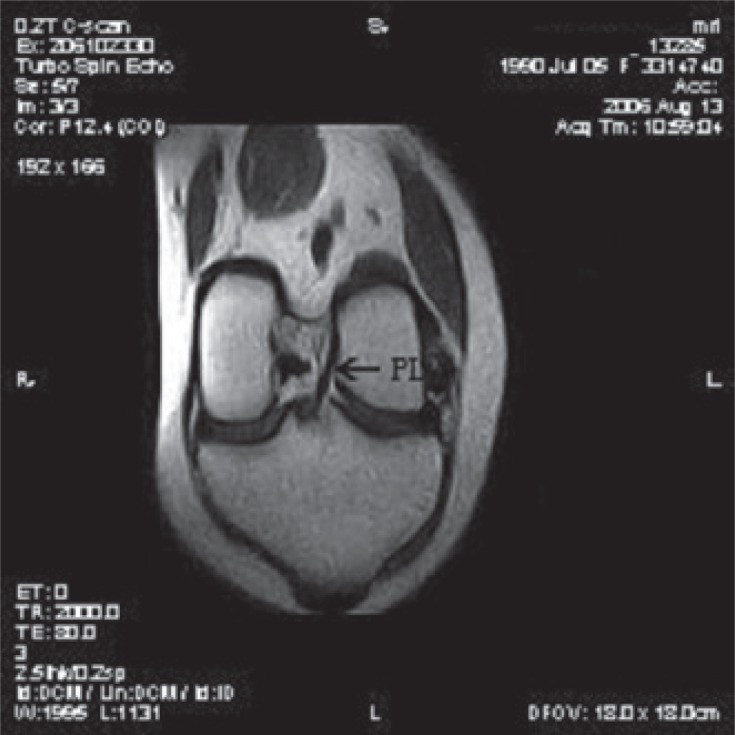
3D reconstruction using the oblique coronal plane showing the posterolateral (PL) bundle.

The oblique coronal slices were suitable for displaying the AM and PL bundles separately. In the imaging slab, the anterior slices displayed the AM and the posterior slices displayed the PL bundles (Figs. 1–3; Table II). In the central image, which showed the two bundles next to each other, a septum-like high intensity structure was observed between the two bundles. The oblique coronal plane visualized the femoral and tibial insertion sites with a greater precision than the sagittal and coronal planes.

**Figure 4.**
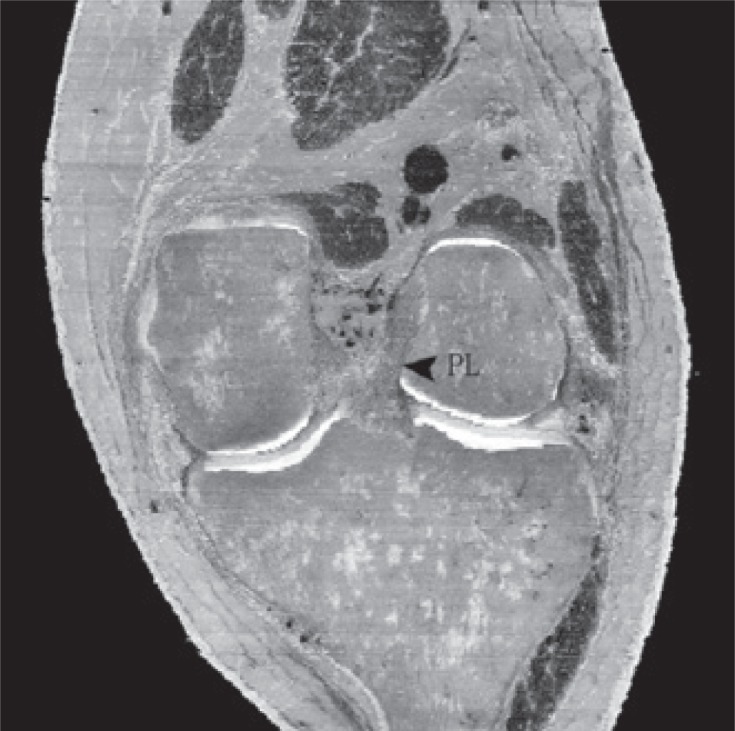
Oblique coronal plane magnetic resonance imaging (MRI) showing the anteromedial (AM) bundle bundle (arrow).

**Figure 5.**
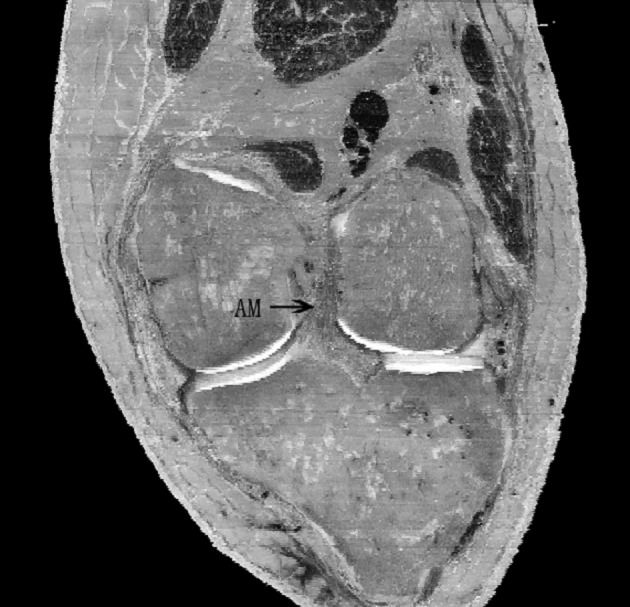
Oblique coronal plane magnetic resonance imaging (MRI) showing the anteromedial (AM) and posterolateral (PL) bundles and a high-intensity fibrofatty septum (arrow).

**Figure 6.**
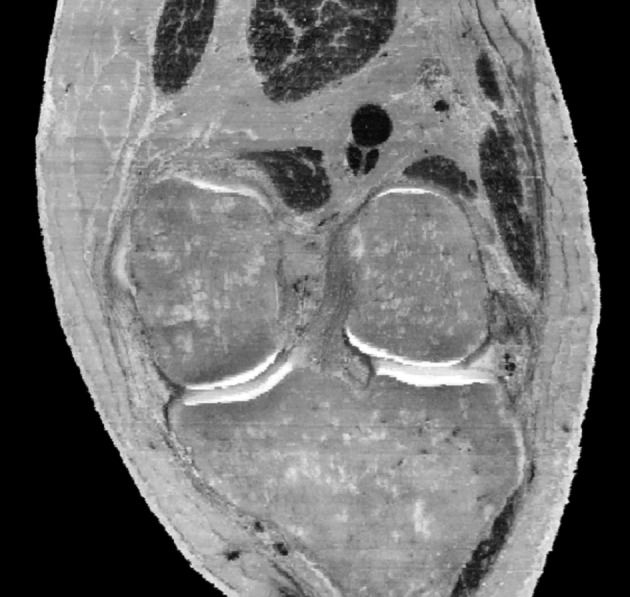
Oblique coronal plane magnetic resonance imaging (MRI) showing the posterolateral (PL) bundle.

In the Chinese Visible Human male dataset, the 3D visualization model of the knee provided an anatomical insight into the ACL in the axial, sagittal and coronal planes; however, the integrity of the AM and PL bundles in their course was not displayed. On the coronal oblique slices oriented parallel to the femoral intercondylar roof inclination, the two bundles were clearly demonstrated. On the reconstructed images in the coronal oblique plane, the ACL exhibited a diagonal course from the posterior superior aspect of the intercondylar surface of the lateral femoral condyle, corresponding to Blumensaat’s line. The length was 35 mm and the thickness 6 mm, running obliquely through the lateral third of the intercondylar notch to the tibial insertion on the central and medial section of the anterior intercondylar area of the tibia. On the reconstructed coronal oblique images, the bony attachment of the ACL was clearly shown on the posterior inner surface of the lateral femoral condyle. The AM bundle was inserted into the cartilage of the ascending section of the central ridge of the medial tibial plateau and the anteromedial tubercle of the intercondylar eminence. The PL bundle was inserted into the cartilage of the ascending section of the central ridge of the lateral tibial plateau, and the posterolateral tubercle of the intercondylar eminence (Figs. 4–6). A septum-like, fatty synovial tissue occupied the space between the two bundles. In the oblique coronal plane, the AM bundle of the ACL was limited distally by the cartilage covering the medial femoral condyle and formed the vertically-oriented section of the PCL complex (Fig. 1).

## Discussion

MRI is the method of choice for imaging the ACL in cases of suspected lesions, particularly in surgical planning. Since the anatomy of this ligament is difficult to capture using traditional slice orientations, the present study optimized the slice orientation adapted to the anatomy of the ligaments. To verify the diagnostic quality of the acquired images, particularly at a low magnetic field strength of 0.2 T in a dedicated musculoskeletal MRI scanner, the resulting images were compared with reconstructed slices from a sectional anatomy dataset: the CVH dataset.

The ACL is one of the principle ligaments that stabilize the knee. From a functional perspective, it is composed of two separate bundles: The AM bundle originates from the superior aspect of lateral side of the femoral notch and inserts into the anteromedial part of tibia, whereas the PL bundle arises from a lower level of the lateral side of the femoral notch and inserts into the posterolateral part of tibia (9–11). The results of the present study were consistent with the double-bundle model. The two bundles are not isometric in flexion and extension, instead exhibiting different patterns of length changes during passive knee flexion (12). The AM of the ACL primarily controls the anterior movement of the tibia underneath the femur, while the PL bundle is significant in maintaining the rotational stability in the knee, such as during pivoting, twisting and jumping (13,14). Hollis *et al* demonstrated that the AM lengthens and tightens in flexion, while the PL shortens and becomes slack (15).

Steckel *et al* (16) evaluated the double-bundle structure of the ACL with 3 T high-field MRI of cadaver knees. The AM and PL bundles were visible in the majority of views, suggesting that a higher field strength may be more effective for distinct imaging. Starman *et al* (10) assessed the standard planes of view for the detection of the AM and PL bundles. The results suggested that standard sagittal and coronal views may allow a reliable detection of the AM bundle; however, the PL bundle is more difficult to image. It is likely that these results were due to a partial volume or blurring effect of the low-resolution images of the two adjacent bundles, making them appear as only one structure.

High-quality visualizations of the individual ACL bundles may be achieved most effectively in planes that are based on the natural course of the ACL (7,17). The ACL has an average angle ranging from 35–40° to the coronal plane. The measured length of the ACL ranges from 32 to 38 mm, with a width ranging from 4 to 7 mm in the oblique sagittal plane. In this plane, it was possible to distinguish the AM and PL bundles.

The current standard in ACL reconstruction surgery is the single-bundle arthroscopic technique (18); however, a number of surgeons have advocated the DBT to restore the AM and PL bundles separately, based on biomechanical and kinematic studies (3). In a partial tear of the ACL, the AM or PL bundle may be intact, and novel surgical techniques enable the preservation of an intact AM or PL bundle (19). The preoperative assessment of the injured ligament is therefore essential for surgeons, as it is necessary to identify whether an intact bundle is present. This leads to a more refined surgical approach and improved outcomes. Complete ACL tears may be accurately identified in each of the standard MRI planes (sagittal, coronal and axial) with high sensitivity and specificity (17,20); however, the identification of partial ACL tears in standard planes using a low-field strength musculoskeletal MRI has been demonstrated to be more challenging (21). The results of the present study showed that the AM and PL bundles of the ACL may be clearly displayed on oblique coronal sections on anatomical 3D reconstructions and low-field MRI. Furthermore, a septum was observed between the AM and the PL bundles, which was not described in previous studies (22,23). The septum-like structure was detected on the present MR images as a high-intensity structure, which may be correlated to fibrofatty and synovial tissue in the CVH 3D model.

Using this method of adapting the MRI planes to the knee anatomy, it may be possible to improve the depiction of structural details of the AM and PL bundles. The additional information gained is likely to improve the diagnostic accuracy in the grading of ACL injuries, which may support the surgical decision for ACL reconstruction. The oblique coronal plane was shown to be the preferred imaging plane for the assessment of the bundle architecture. This result was consistent with the study by Hong *et al* (7), which demonstrated that the diagnostic accuracy for ACL tears was improved with additional oblique coronal planes compared with the standard orientations.

High-resolution isotropic datasets from cryosections and MRI may be reformatted using visualization software on standard PC hardware. With this technique, images of the knee, and particularly the ACL, may be reconstructed in virtually every orientation, in the conventional axial, coronal and sagittal planes, as well as in the oblique coronal plane. The anatomical view of the ACL shown with the oblique coronal plane is most suitable for displaying the shape of the ACL and its anatomical relationships. The results of the present 3D cryosectional and MRI visualization study were comparable with the observations by Stäubli and Rauschning (24), obtained with contrast magnetic resonance arthrography. The anatomical orientation of the oblique course of the ACL is parallel to Blumensaat’s line. In the present study, a triangle was observed that was formed by the AM and PL bundles and their tibial attachments and filled by fibrofatty and synovial tissue. The MRI observations of the ACL were consistent with these results. The 3D reconstruction revealed that the two bundles were not isometric in the oblique coronal plane. It was also demonstrated that the long axes of the two bundles were not parallel.

Based on evidence from the 3D visualization of the CVH dataset and MRI analysis of the cruciate ligaments in the oblique coronal plane, oblique coronal imaging of the ACL in extension may be recommended for inclusion in routine MR knee imaging protocols for the assessment of ACL tears, particularly if the diagnosis of an ACL tear is doubtful, based on clinical findings. Multiplanar reconstruction and the use of the oblique coronal plane may improve the understanding of the ACL anatomy and provide a valuable method for teaching and research. The reconstructed images provide the anatomical basis for imaging diagnosis and surgery.

This study used a low-field strength musculoskeletal MRI scanner and 3D visualization in oblique coronal planes for the assessment of the double-bundle anatomy of the ACL. The results showed that this technique reliably visualizes the two bundles of the ACL. The use of additional oblique slices for the evaluation of the AM and PL bundles offers a potential solution for an improved evaluation with low-field strength magnets. It may assist in the preoperative assessment and grading of ACL injuries to facilitate ACL reconstruction, in addition to being used in research and teaching.

## Figures and Tables

**Table I.** Measurement of the ACL in the oblique plane (n=60).

Group	Length (mm)	Thickness (mm)	Angle (°)
Left knee	35.31±1.76	5.79±1.08	36.3±2.3
Right knee	35.66±2.53	5.75±1.21	35.5±2.8
Male	36.45±1.98	6.09±1.07	36.4±2.5
Female	34.52±1.93	5.45±1.13	35.4±2.5

ACL, anterior cruciate ligament.

**Table II.** Agreement between readers with regard to the ability to distinguish the AM and PL bundles of the ACL separately.

Images	Readers		

	1 vs. 2	2 vs. 3	1 vs. 3
Oblique oblique images	0.88	0.91	0.93
Oblique coronal images	1	1	1

AM, anteromedial; PL, posterolateral; ACL, anterior cruciate ligament.
